# Application of Surgical Adhesive Reduces Ureteroureterostomy Leakage

**DOI:** 10.7759/cureus.78866

**Published:** 2025-02-11

**Authors:** Jesus O Tamayo, Michael L Wade, Rylan Fowers, Robert T Mallet, Albert H Yurvati

**Affiliations:** 1 Physiology and Anatomy, Texas College of Osteopathic Medicine, Fort Worth, USA; 2 Physiology and Anatomy, University of North Texas Health Science Center, Fort Worth, USA; 3 Surgery, Texas College of Osteopathic Medicine, Fort Worth, USA

**Keywords:** anastomosis, leakage, surgical adhesive, ureter, ureteral injury, ureteral repair, ureteroureterostomy

## Abstract

Their close proximity to abdominal structures and organs places the ureters at risk of iatrogenic injury during abdominal and pelvic surgical procedures. Ureteroureterostomy is the mainstay intervention for the repair of the proximal and middle ureter. However, the anastomosis may be subject to leakage, leading to potentially serious complications, including urinary tract infection, peritonitis, sepsis, and kidney damage. This study addressed the hypothesis that topical application of surgical adhesive reduces acute anastomotic leakage without increasing ureteric resistance. Nine ureters from domestic swine were pump-perfused with 0.9% sodium chloride (NaCl) at approximately 5, 10, 12, 15, and 20 mmHg (2 min/step), while flow and ureteral resistance were monitored, under three conditions: pre-transection, following ureteroureteral anastomosis with suture, and after topical application of Dermabond® surgical adhesive around the anastomosis circumference. Leakage was taken as the loss of fluid volume between the perfusate reservoir and the post-ureter receptacle. Values were compared by two-factor repeated measures ANOVA combined with the post hoc Tukey test. Leakage from the perfused ureter increased severalfold following transection and surgical anastomosis vs. pre-transection at all five perfusion pressures. Application of surgical adhesive to the anastomosis returned leakage to the pre-transection rate across the entire perfusion pressure range. There were no significant differences in ureteral flows and resistances among the three conditions. Thus, the application of surgical adhesive to the ureteroureteral anastomosis effectively prevented ureteric leakage at physiologic luminal pressures without increasing ureteral flow resistance.

## Introduction

The ureters are delicate bilateral conduits that transfer urine from the renal pelvis to the bladder trigone. Each ureter courses through the retroperitoneal space in close proximity to several abdominal and pelvic structures, including the iliac and uterine arteries, cervix, vagina, and rectosigmoid colon [1,2]. Their location places the ureters at risk of iatrogenic injury during diagnostic or medical procedures targeting the abdominal and pelvic organs [3]. Approximately 75% of ureteric injuries (UIs) are iatrogenic [4,5], most commonly in urologic, gynecologic, and colonic surgeries [6]. Typical iatrogenic UIs include ureteral transection, kinking, suture-imposed obstruction, and stricture [5]. A kidney transplant requires graft ureter transection and anastomosis to the recipient’s ureter or bladder [7].

Common UI complications include urinary tract infections, peritonitis, ureter fistula, and kidney damage [8-10]. Surgical repair is the mainstay intervention, with the position of the lesion being the primary determinant of the repair method [6]. While ureteroneocystostomy is the intervention of choice for UIs within 4-6 cm of the ureterovesical junction, ureteroureterostomy is better suited for more proximal damage [11]. The “lid technique” is utilized to effect vascular and ureteral end-to-end anastomosis [12,13]. Complications of suboptimal UI repair include hydronephrosis due to ureteric stricture and peritonitis due to urine leakage into the retroperitoneum [6,14]. Anastomosis leakage can lead to abdominal sepsis and pyelonephritis [15]. Treatment of ureteral leak may be conservative with supportive care, or more invasive with temporary ureteral stenting [16].

While the use of surgical adhesive in combination with suture has proven to prevent leakage of vascular anastomosis [17,18], its use in urologic procedures, such as ureteral repair, is not well documented. Few studies have investigated the use of fibrin adhesive in suture and reduced suture porcine ureter repair in vivo [19,20]. They showed that mortality was unaffected and there was no stenosis after 18 months [19]. The use of fibrin adhesive without stay sutures may increase the risk of urinoma. Appropriate application of surgical adhesive to the ureteroureteral anastomosis could prevent urinary leakage and its complications while decreasing the time for ureteral repair. Accordingly, this study tested the hypothesis that ureteroureterostomy reinforcement with surgical adhesive decreases leakage at the surgically repaired ureteral anastomosis. The impacts of suture alone vs. suture reinforced with adhesive on ureteral patency, resistance, and anastomosis integrity were evaluated at intraureteric pressures of 5-20 mmHg, encompassing the physiological pressure range [21] and elevated pressures during ureteroscopy [22].

## Materials and methods

Ethical approval

Ethical approval for this study was received from the University of North Texas Health Science Center’s (UNTHSC) Institutional Animal Care and Use Committee (IACUC) and all experimentation was performed at the University of North Texas Health Science Center.

Ureter perfusion system

**Figure 1:**
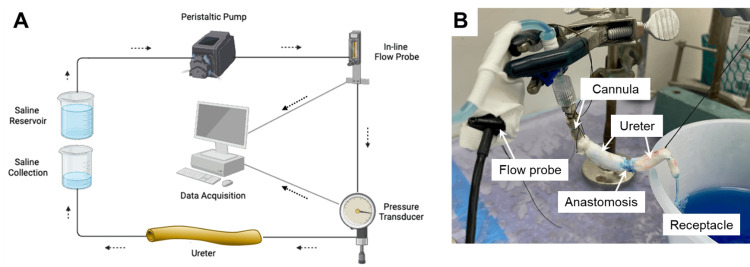
Ureter perfusion system. The system is diagramed in panel A (image created by the author using BioRender.com). Panel B: Photograph of the perfused ureter experiment. Blue dye was added to the perfusion medium to visualize the perfusate.

This study employed a perfusion system (Figure 1) consisting of an adjustable phasic pump (ISMATEC Ecoline, ISM1076, Glattbrugg, Switzerland), pressure/flow servo-regulator (emKa Technologies, Sterling, VA), in-line flow probe (ME 3 PXN, Transonic Systems, Ithaca, NY), and pressure transducer (emKa Technologies, Sterling, VA). The flow probe and pressure transducer enabled control of ureteral perfusion pressure by adjusting inflow. Pressure and flow data were acquired continually on a personal computer equipped with the Spike2 data acquisition system (Cambridge Electronic Design Limited, Cambridge, UK).

Surgical preparation of ureters

Ureters were obtained from four male and three female Yorkshire swine (mass: 35-60 kg). After an overnight fast, the pigs were sedated with an intramuscular cocktail of Telazol® (5 mg/kg) and xylazine (2 mg/kg). After sedation and intubation, a surgical plane of anesthesia was maintained throughout the experiment by spontaneous respiration with 1-5% isoflurane supplemented with 1-2 L/min O_2_.

The pigs were placed supine on a heated pad on the surgery table and the body temperature monitored with a rectal thermal probe. After aseptic preparation of the surgical site, an abdominal incision was performed. Kidney vasculature was skeletonized to the aorta and inferior vena cava. The ureters were followed and dissected from the renal pelvis to its insertion at the bladder. Approximately 20 cm of ureter and periureteral tissue were preserved. Both ureters were obtained from two pigs (male), in which the left and right kidneys were excised sequentially, along with the respective renal arteries, veins, and ureters. In five pigs (two males and three females), only the right ureter was harvested for this study. The renal artery was cannulated, the kidney was flushed for 15 minutes with c. 400 ml University of Wisconsin preservation solution, and then the ureter was excised at the ureteropelvic junction and prepared for perfusion.

Ureter perfusion

An approximately 10 cm segment of the distal ureter was isolated, cannulated, connected to the perfusion system (Figure 1B), and perfused with 0.9% saline at target pressures of 5, 10, 12, 15, and 20 mmHg for 120 seconds at each pressure. These perfusion pressures encompassed and exceeded the physiological pressure range of 5-10 mmHg reported in human ureters [21].

After completing the initial (baseline) perfusion sequence, the ureter and cannula were disconnected from the perfusion system, and the ureter was transected at its midpoint with a scalpel. The two segments were anastomosed by the lid technique [12] and modified by the use of a continuous suture. In accordance with the lid technique, 1 cm longitudinal incisions were made in each stump of the transected ureter, on opposite sides of the ureteric circumference. The anastomosis was effected with a continuous suture around the circumference of the transection, locking every third throw to ensure a tight seal. Approximately 15 throws were used, and the suture was tied to its origin with care taken to avoid creating iatrogenic strictures. To maintain patency and avoid stricture during suturing, a 2 mm diameter capillary tube was inserted into the transected ends of the ureter segments, which were then approximated with a Deknatel® 3-0 needle with braided polyester fiber suture (Teleflex Inc., Wayne, PA).

After transection and anastomosis, the repaired, cannulated ureter was reattached to the perfusion system, and the 5-20 mmHg perfusion pressure sequence was repeated. Next, the ureter was again disconnected from the perfusion system, and approximately 80-120 milligrams (3-5 drops) of Dermabond Advanced™ topical adhesive (active ingredient: 2-octyl cyanoacrylate; Ethicon Inc., Raritan, NJ) was evenly applied around the full circumference of the anastomosis site and allowed to anneal for five minutes. The capillary tube was reinserted and advanced to the transection site during annealing and then removed before reconnecting the ureter to the perfusion system. The cannulated ureter was reattached to the perfusion system, and the 5-20 mmHg perfusion protocol was performed a third time.

Calculations

The masses (g) of 0.9% sodium chloride (NaCl) in the source reservoir and collection receptacle were measured before and after each 120-second perfusion at the designated pressures. Because the ureter outlet was open to the atmosphere, the flow resistance of the perfused ureter equaled perfusion pressure (mmHg) divided by flow (ml • min^-1^). The fluid volume lost between the reservoir and receptacle was computed by the following equation:

$\frac{(Initial - Final \ Reservoir \ Mass) - (Final - Initial \ Receptacle \ Mass)}{1.0049 g/ml} = fluid \ loss \ (ml)$

Where the density of 0.9% NaCl, i.e., 1.0049 g/ml, was empirically determined on an analytical balance. The incremental increase in fluid volume loss after anastomosis vs. pre-transection baseline was taken as fluid leakage at the anastomosis site.

Statistical analysis

Values were presented as mean ± SEM and analyzed using GraphPad Prism (GraphPad Software, San Diego, CA). Figures were created using GraphPad Prism. Statistical comparison of ureter flows, resistances, and leakage rates during the pre-transection baseline, suture, and suture + adhesive (n = 9) were compared by two-factor (pressure, treatment) ANOVA with repeated measures on both factors. When ANOVA detected a statistically significant treatment effect (P < 0.05), a post-hoc Tukey test was performed to identify pairwise differences between treatments. For comparison of anastomosis leaks with vs. without adhesive, a two-factor ANOVA with repeated measures on both factors with a post-hoc Šídák's test was used.

## Results

Ureteral perfusion pressures

Ureteral flows and resistances were determined at perfusion pressures of 5, 10, 12, 15, and 20 mmHg, for 120 seconds at each pressure. The measured perfusion pressures approximated the target pressures across the entire pressure range and were nearly identical during the three pressure runs, i.e., pre-transection baseline, following ureteral transection and anastomosis, and after application of surgical adhesive to the anastomosis (Table 1).

**Table 1:** Ureteral perfusion pressures. Values (means ± SEM) represent the mean pressures over 120 seconds of perfusion at each target pressure. There were no statistically significant differences among the three conditions.

Target perfusion pressure	Measured perfusion pressure (mmHg)		
	Baseline	Suture	Suture and adhesive
5 mmHg	4.82 ± 0.13	4.80 ± 0.12	4.85 ± 0.16
10 mmHg	9.83 ± 0.10	9.77 ± 0.12	9.85 ± 0.08
12 mmHg	11.80 ± 0.08	11.88 ± 0.13	11.83 ± 0.05
15 mmHg	14.73 ± 0.06	14.81 ± 0.10	14.94 ± 0.08
20 mmHg	19.77 ± 0.14	19.80 ± 0.19	19.95 ± 0.11

Impact of ureteral anastomosis ± surgical adhesive on ureter flow and resistance

**Figure 2:**
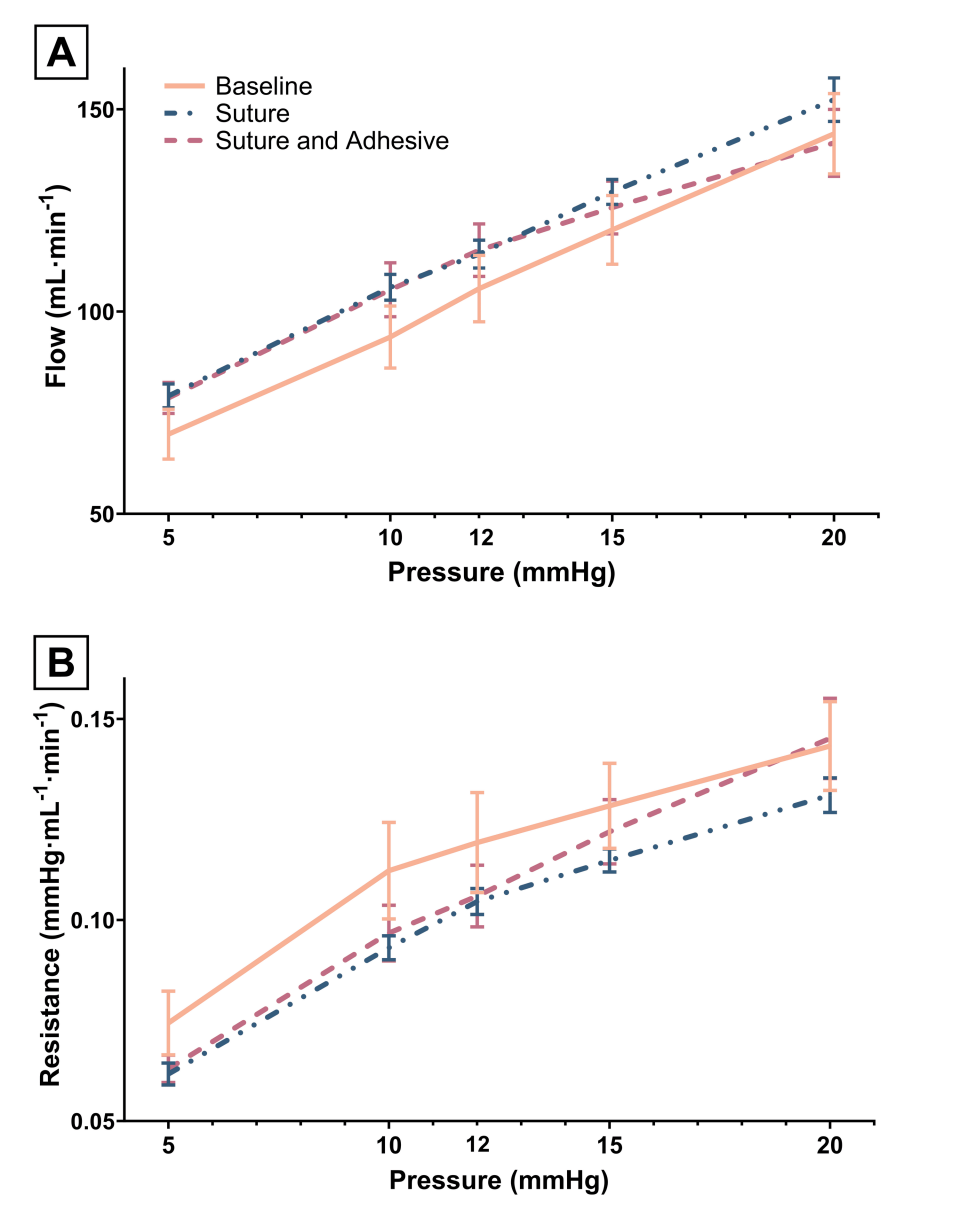
Ureter flow and resistance at 5-20 mmHg perfusion pressure. Flows (panel A) and perfusion pressures were measured at the proximal end of the cannulated ureter before ureteral transection (baseline), after ureteral anastomosis with suture (suture only), and after application of surgical adhesive (suture and adhesive). Resistance (panel B) was computed as pressure (mmHg)/flow (ml/min). Mean values ± SEM are depicted.

Before transection, ureteral flow increased approximately linearly with perfusion pressure (Figure 2A), while resistance increased more sharply between 5 and 10 mmHg than at higher perfusion pressures (Figure 2B). Ureteral transection and anastomosis with suture alone produced a modest, albeit not statistically significant upward shift in flow and a commensurate decrease in resistance. Application of the adhesive limited the increases in flow at 15 and 20 mmHg, while resistance increased more sharply at these perfusion pressures than in the other two conditions.

Surgical adhesive effectively prevents anastomosis leakage

**Figure 3:**
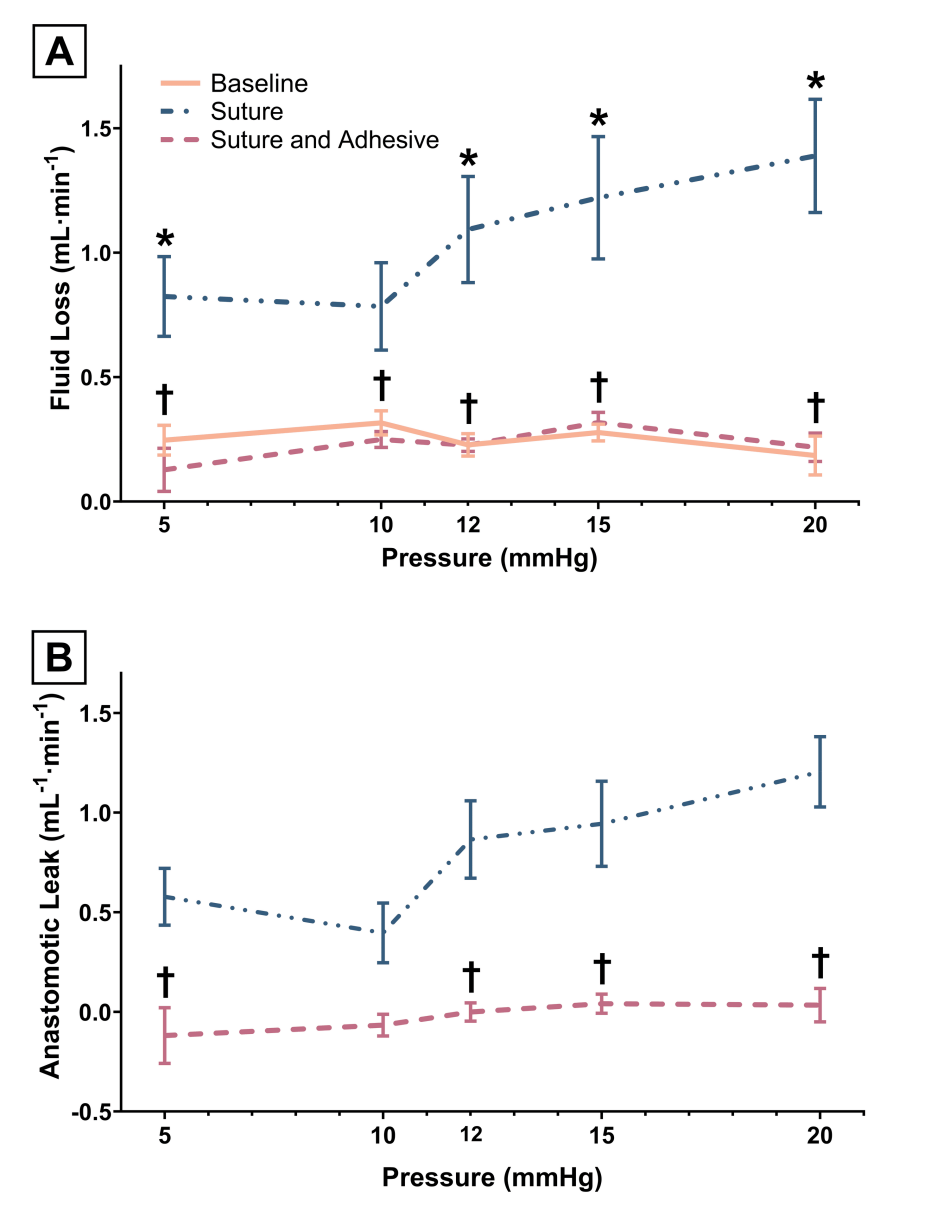
Ureteral fluid leakage. The loss of fluid volume between the proximal and distal ends of the perfused ureters was determined over two-minute perfusion at pressures between 5 and 20 mmHg (panel A). Subtraction of the baseline fluid loss of the non-transected ureters yields fluid loss ascribable to leakage at the anastomosis (panel B). Values are presented as mean ± SEM. * P < 0.05 vs. baseline; † P < 0.05 vs. suture only.

Before transection, fluid loss from the perfused ureters was limited and did not change with increasing perfusion pressures (Figure 3A, baseline). Fluid leakage increased sharply following ureteral transection and anastomosis (Figure 3A, suture), particularly at the upper end of the perfusion pressure range. Application of surgical adhesive to the anastomosis returned fluid leakage to the baseline rate at all perfusion pressures (Figure 3A, suture and adhesive), and fluid loss after application of adhesive differed (P < 0.05) from suture alone over the entire perfusion pressure range. Furthermore, when compared to baseline, repaired transection with suture and adhesive had no significant difference over the full perfusion pressure range. Thus, the surgical adhesive eliminated leakage from the ureteral anastomosis at perfusion pressures up to 20 mmHg. When baseline, i.e., transection-independent, fluid losses were subtracted from total losses (Figure 3B), the adhesive reduced significantly the rate of anastomosis leak at 5, 12, 15, and 20 mmHg.

## Discussion

Ureteral injuries are potentially serious complications of colorectal and gynecological surgery as well as penetrating abdominal trauma. Although laparoscopic ureteral anastomosis is the mainstay treatment for moderate to severe ureteral injury, ureteric leakage and stricture may prolong recovery or worsen morbidity. Anastomosis leakage can lead to urinoma, intra-abdominal abscess, and pyelonephritis [15]. While less common, sepsis is a potentially lethal complication of ureteral injury and unsuccessful reconstruction [23]. Ureteral stricture at the anastomosis site is the predominant long-term complication of ureter repair. Anastomotic stricture increases pressure proximal to the constriction, imposing a heightened risk of hydronephrosis, renal failure, and hypertension [14,24].

This study demonstrated the application of surgical adhesive to the sutured anastomosis effectively prevented leakage at ureteral pressures encompassing and exceeding the physiologic range [21]. Additionally, an increase in luminal pressure produced by distal urolithiasis or ureteroscopy [22] could exacerbate anastomotic leakage, as seen in the suture-only condition. Importantly, the use of surgical adhesive prevented leakage, even at higher pressures.

By limiting surgical complications, the use of adhesive for ureteral reconstruction may reduce the duration of post-surgical hospital stay [25,26]. BioGlue® is currently the only FDA-approved surgical adhesive for cardiac and vascular repair surgeries as an adjunct to staples or sutures [27]. Further study of this adhesive in ureter repair is essential to secure regulatory approval for that indication.

The long-term impact of surgical adhesive on ureteral patency and healing of the anastomosis merits investigation. The impact of surgical adhesive on ureteral healing is not yet known. Previous studies investigating ureter repair with surgical adhesive indicate that the use of fibrin adhesive may decrease the risk of stricture formation [19]. While the use of adhesive alone for ureter repair has been investigated previously [20,28], this study shows that reinforcement of suture with adhesive can increase the integrity of the anastomosis, even at supraphysiological pressures. Studies in vascular anastomoses show little or no adverse effect of surgical adhesive on anastomotic integrity or vascular healing and function [17,18]. If vascular surgery findings can be extrapolated to ureteral anastomoses, they suggest surgical adhesive is unlikely to impede the healing and re-integration of the ureteral segments at the anastomosis.

While minor anastomosis leakage typically resolves spontaneously, ureteric stricture, a not uncommon complication of ureteral reconstruction [5,6], may result from post-surgical infection, inflammation, and surgery [29]. In this study, the use of an intraluminal capillary tube kept the ureter patent while the adhesive dried. Employing such a strategy in clinical settings could minimize touching of the luminal surface of the ureter during anastomosis, a risk factor for increased scar tissue and stricture development [29].

The application of surgical adhesive tended to flatten the ureteral pressure and flow relationship and increase ureteral resistance at the higher end of the perfusion pressure range. Although not statistically significant, these findings suggest the annealed surgical adhesive may have restricted ureteral expansion at the anastomosis site at supraphysiologic pressures. This phenomenon may be important in patients with a history of nephrolithiasis, where limited ureter dilation may complicate the condition [30].

In addition to limiting or preventing anastomotic leakage, the use of surgical adhesive could permit more efficient use of suture material during repair. Preservation of periureteral blood supply is an important prognostic factor for anastomosis success [31]. Excessive suturing may reduce nutritive blood flow to the ureteral smooth muscle [32], delaying healing. Future studies should investigate the use of surgical adhesive in minimizing suturing to improve intramural blood flow and facilitate healing.

Strengths and limitations of the study

Strengths of the study include the use of domestic swine, a well-accepted large animal model of the human urinary system, as the source of ureters. Indeed, the dimensions [33] and biomechanical properties [34] of porcine ureters strongly resemble those of human ureters. The study’s repeated measures design, in which each ureter was tested before transection and in the absence and presence of adhesive, minimized variations in ureter properties among the three pressure-flow protocols.

The limitations of this study are acknowledged. The porcine ureters were repaired and evaluated at room temperatures ex vivo, which may produce different results than an in vivo experiment. The ex vivo ureter was fully exposed to air, which may accelerate the annealing process. Also, cyanoacrylate-based Dermabond®, which is used for wound closure and superficial lacerations, was used instead of an internal adhesive. Cyanoacrylate adhesives may elicit inflammation, tissue necrosis, or foreign body reaction [27] and repeated exposure to cyanoacrylates is potentially toxic to medical professionals [28]. Although the use of adhesive may introduce cyanoacrylate into the lumen of the reconstructed ureter, the suture and lid technique may limit this problem. Future studies should investigate the use of internal adhesives in vivo to evaluate the practicality of using such adhesives for ureter repair. Finally, only the acute effects of the newly applied adhesive were examined. The long-term impacts of surgical adhesive on post-surgical ureteric healing and anastomosis integrity merit investigation.

## Conclusions

In this study, transected porcine ureters anastomosed with suture demonstrated appreciable fluid leak at the anastomosis when saline-perfused at physiological luminal pressures. The anastomotic leakage increased further at supraphysiological fluid pressures. Topical application of surgical adhesive to the anastomosis stopped the leakage at physiological and supraphysiological luminal fluid pressures without increasing appreciably ureteric flow resistance. Surgical adhesive may be an effective adjunct to ensure complete anastomosis closure in surgically repaired ureters, thereby preventing the adverse effects of retroperitoneal urine leakage.

## References

[1] Jackson LA, Ramirez DM, Carrick KS, Pedersen R, Spirtos A, Corton MM (2019). Gross and histologic anatomy of the pelvic ureter: clinical applications to pelvic surgery. Obstet Gynecol.

[2] Fröber R (2007). Surgical anatomy of the ureter. BJU Int.

[3] Elawdy MM, Osman Y, Awad B, El-Mekresh M, El-Halwagy S (2021). Iatrogenic ureteral injuries: a case series analysis with an emphasis on the predictors of late ureteral strictures and unfavorable outcome in different surgical specialties. Int Urogynecol J.

[4] Ali MA, Maalman RS, Oyortey MA, Donkor YO, Adanu KK, Tampuori J, Kyei MY (2022). A 6-year retrospective clinical review of iatrogenic ureteric injuries repaired in a resource-deprived setting. BMC Surg.

[5] Esparaz AM, Pearl JA, Herts BR, LeBlanc J, Kapoor B (2015). Iatrogenic urinary tract injuries: etiology, diagnosis, and management. Semin Intervent Radiol.

[6] Burks FN, Santucci RA (2014). Management of iatrogenic ureteral injury. Ther Adv Urol.

[7] Suttle T, Fumo D, Baghmanli Z, Saltzman B, Ortiz J (2016). Comparison of urologic complications between ureteroneocystostomy and ureteroureterostomy in renal transplant: a meta-analysis. Exp Clin Transplant.

[8] Momin AA, Barksdale EM 3rd, Lone Z (2020). Exploring perioperative complications of anterior lumber interbody fusion in patients with a history of prior abdominal surgery: a retrospective cohort study. Spine J.

[9] Vorobev V, Beloborodov V, Golub I, Frolov A, Kelchevskaya E, Tsoktoev D, Maksikova T (2021). Urinary system iatrogenic injuries: problem review. Urol Int.

[10] Piriyev E, Schiermeier S, Römer T (2022). Are double-J stents in surgery for deep infiltrating endometriosis always necessary? A retrospective analysis. Wideochir Inne Tech Maloinwazyjne.

[11] Tyagi V, Jain S, Singh M, Pahwa M, Chadha S, Rasool S (2019). Native ureteroureterostomy in renal allograft recipient surgery: a single-center 5-year experience. Indian J Urol.

[12] Gürhan Ulusoy M, Kankaya Y, Uysal A, Sungur N, Koçer U, Kankaya D, Oztuna D (2009). "Lid technique": cyanoacrylate-assisted anastomosis of small-sized vessels. J Plast Reconstr Aesthet Surg.

[13] Elliott SP, McAninch JW (2006). Ureteral injuries: external and iatrogenic. Urol Clin North Am.

[14] Lucarelli G, Ditonno P, Bettocchi C, Grandaliano G, Gesualdo L, Selvaggi FP, Battaglia M (2013). Delayed relief of ureteral obstruction is implicated in the long-term development of renal damage and arterial hypertension in patients with unilateral ureteral injury. J Urol.

[15] Herson AB, Villacreses CA, Rehm GM (2023). Conservative management of postoperative urinary leak and intra-abdominal abscess. Cureus.

[16] Park HJ, Shin JH, Kim JW, Hong BS (2015). Postoperative ureteral leak treated using a silicone-covered nitinol stent. Int Neurourol J.

[17] Ribaudo JG, He K, Madira S (2024). Sutureless vascular anastomotic approaches and their potential impacts. Bioact Mater.

[18] Schulten L, Spillner J, Kanzler S, Teubner A, Jockenhoevel S, Apel C (2022). A polyurethane-based surgical adhesive for sealing blood vessel anastomoses—a feasibility study in pigs. J Biomed Mater Res B Appl Biomater.

[19] Anidjar M, Desgrandchamps F, Martin L, Cochand-Priollet B, Cussenot O, Teillac P, Le Duc A (1996). Laparoscopic fibrin glue ureteral anastomosis: experimental study in the porcine model. J Endourol.

[20] Detweiler MB, Detweiler JG, Fenton J (1999). Sutureless and reduced suture anastomosis of hollow vessels with fibrin glue: a review. J Invest Surg.

[21] Shabayek M, Osman T, Wahb M, Elmoazen M, Osman D, Saafan A (2022). Intravesical aminophylline instillation as an alternative for balloon dilatation prior to semi-rigid ureteroscopic management of distal ureteral stones. World J Urol.

[22] Schwalb DM, Eshghi M, Davidian M, Franco I (1993). Morphological and physiological changes in the urinary tract associated with ureteral dilation and ureteropyeloscopy: an experimental study. J Urol.

[23] Fry DE, Milholen L, Harbrecht PJ (1983). Iatrogenic ureteral injury. Options in management. Arch Surg.

[24] Wiesner C, Thüroff JW (2004). Techniques for uretero-intestinal reimplantation. Curr Opin Urol.

[25] Uccelli M, Targa S, Cesana GC (2021). Use of fibrin glue in bariatric surgery: analysis of complications after laparoscopic sleeve gastrectomy on 450 consecutive patients. Updates Surg.

[26] Mercier G, Loureiro M, Georgescu V (2017). Surgical glue in laparoscopic sleeve gastrectomy: an initial experience and cost-effectiveness analysis. J Eval Clin Pract.

[27] Sanders L, Nagatomi J (2014). Clinical applications of surgical adhesives and sealants. Crit Rev Biomed Eng.

[28] Leggat PA, Smith DR, Kedjarune U (2007). Surgical applications of cyanoacrylate adhesives: a review of toxicity. ANZ J Surg.

[29] Mundy AR, Andrich DE (2011). Urethral strictures. BJU Int.

[30] Moynihan MJ, Mandeville JA, Flacke S, Moinzadeh A (2020). A novel technique of ureteral stricture measurement: impact on diagnosis and subsequent management. J Endourol Case Rep.

[31] Rossanese M, Giannarini G, Scalia R, Macchione L, Crestani A, Simonato A, Ficarra V (2023). Outcomes and treatment failure after open or robotic ureteral reconstruction for iatrogenic injuries. BJUI Compass.

[32] Pacer E, Griffin DW, Anderson AB, Tintle SM, Potter BK (2020). Suture and needle characteristics in orthopaedic surgery. JBJS Rev.

[33] Smit JH, Leonardi EP, Chaves RH, Furlaneto IP, Silva CM, Abib SC, Góes Junior AM (2021). Image-guided study of swine anatomy as a tool for urologic surgery research and training. Acta Cir Bras.

[34] Casarin M, Toniolo I, Todesco M, Carniel EL, Astolfi L, Morlacco A, Moro FD (2024). Mechanical characterization of porcine ureter for the evaluation of tissue-engineering applications. Front Bioeng Biotechnol.

